# Case Report: Allergic reaction following fecal microbiota transplantation in children with autism spectrum disorder: a report of two cases

**DOI:** 10.3389/fped.2026.1847568

**Published:** 2026-06-16

**Authors:** Zhihui Peng, Yuanbing Hu, Sheng Wang, Chunhua Liu, Qi Zhou, Shiyu Xie, Silin Huang, Nan Liu, Yanli Wang

**Affiliations:** 1 Department of Pediatrics, South China Hospital, Medical School, Shenzhen University, Shenzhen, China; 2 Department of Gastroenterology, South China Hospital, Medical School, Shenzhen University, Shenzhen, China; 3 Institute of Environment and Health, South China Hospital, Medical School, Shenzhen University, Shenzhen, China

**Keywords:** anaphylactic shock, autism spectrum disorder, fecal microbiota transplantation, food intolerance, side effect

## Abstract

**Background:**

Fecal microbiota transplantation (FMT) is increasingly used in children with autism spectrum disorder (ASD) and is generally considered to have an acceptable safety profile. However, the risk and mechanisms of severe adverse events in the high-risk subgroup of children with ASD complicated by multiple food intolerances (FI) have not been reported, and clinical strategies for risk prevention and management remain lacking.

**Case report:**

This article reports two cases of children with ASD and multiple FIs who developed anaphylactic shock following FMT, representing the first such report in the pediatric field. Case 1 presented with early-stage shock, which was considered to be associated with IgG-mediated type III hypersensitivity. Case 2 developed classic anaphylactic shock with a biphasic course characterized by relapse after initial resolution, raising the possibility of biphasic anaphylaxis. Both children improved after standardized anti-allergy and anti-shock treatment, and one child successfully completed subsequent FMT using a low-dose regimen.

**Conclusion:**

FMT carries a risk of anaphylactic shock in children with ASD and multiple FIs, who have underlying susceptibility due to impaired intestinal barrier function and immune dysregulation. Refined risk stratification, close monitoring throughout the procedure, individualized transplantation protocols, and standardized emergency procedures can effectively enhance the safety and feasibility of FMT in this high-risk subgroup.

## 1 Introduction

Autism spectrum disorder (ASD) is a neurodevelopmental disorder characterized by core features including social communication deficits, repetitive behaviors, and restricted interests (1). Studies have shown that children with ASD frequently present with gut microbiota dysbiosis and gastrointestinal symptoms, and this dysfunction of the microbiota–gut–brain axis may exacerbate core symptoms through immune, metabolic, and neuroendocrine pathways (2).

Fecal microbiota transplantation (FMT), as an approach to remodeling the gut microbiota, has preliminary evidence suggesting that it may improve gastrointestinal symptoms and behavioral manifestations in children with ASD, and is considered to have a favorable safety profile (3, 4). Food intolerance (FI) in children is a clinically common non-immune or mixed mechanism-mediated adverse reaction to food, characterized by complex pathogenesis and diverse clinical manifestations involving multiple pathophysiological mechanisms such as intestinal barrier dysfunction, immune homeostasis imbalance, microbial dysbiosis, and enzyme deficiencies (5, 6). Notably, gut microbiota dysbiosis can trigger intestinal immune inflammatory responses, disrupt intestinal mucosal integrity, and facilitate the entry of gastrointestinal hormones and metabolites into the circulation, thereby affecting neuroendocrine function (7). FI is a common comorbidity in children with ASD (8).

This report presents two cases of children with ASD complicated by multiple FIs who were admitted to the Department of Pediatrics, South China Hospital, Shenzhen University, and developed anaphylactic shock following FMT. To the best of our knowledge, this is the first report of pediatric patients developing anaphylactic shock after FMT. These cases highlight the potential risks of FMT in specific populations and aim to enhance clinicians’ awareness and ability to recognize and manage FMT-related adverse events.

**Table 1:** Timeline of severe adverse events following FMT in two children with ASD and food intolerance.

Time point	Case 1	Case 2
Initial visit	February 2025	August 2025
Pre-FMT preparation	Avoidance of intolerant foods; bowel preparation (liquid diet 1 day prior, 10-h food fast/4-h water fast, oral polyethylene glycol electrolyte powder)	Same as left
FMT procedure	Nasojejunal tube placement under colonoscopy, fixed with two hemostatic clips; 50 mL bacterial suspension infused, followed by 10 mL warm saline flush; right lateral decubitus position for 30 min; once every other day for a total of 3 transplantations	Same as left
Time to first symptom after FMT	3 h	10 h
Early manifestations	Apathy, facial flushing, pruritus on both cheeks and ears, abdominal bloating; increased respiratory and heart rates with normal blood pressure	generalized urticarial rash with pruritus; rash resolved 1 h after oral cetirizine 10 mg
Progression to shock	Early-stage shock suspected (blood pressure remained normal)	12 h after FMT: dizziness, severe abdominal pain, vomiting (×6), watery diarrhea (×1); respiratory rate 40/min, pulse 132/min, BP 71/46 mmHg, CRT 5 s → anaphylactic shock
Resuscitation measures	Rapid IV fluid resuscitation (normal saline 10 mL/kg over 15 min); oral cetirizine 2.5 mg	ECG monitoring, oxygen; IV fluid resuscitation (normal saline 20 mL/kg over 10 min); intramuscular epinephrine 1:1,000 (0.17 mg) × 2 doses; IV methylprednisolone sodium succinate (2 mg/kg)
Time to complete symptom resolution	Within 24 h	Within 24 h
Subsequent treatment adjustment	FMT paused, observed for 1 day; switched to low-dose regimen (25 mL per administration every 12 h for 4 times) – no recurrence of adverse events	FMT course not completed; hospitalized for 3 days then discharged
Repeat laboratory findings	Normal CBC, CRP, negative IgE	Normal CBC, CRP, positive total IgE
Follow-up at 1 month	Reduced abdominal pain, bloating, and diarrhea after consuming eggs and milk	No recurrence of allergic symptoms
Repeat FMT	Second FMT in May 2025 – no adverse events	Not performed
Change in ASD scale scores	CARS: 35 → 30; ABC: 55 → 50; improvement in core symptoms	Not available

## 2 Case presentation

Bowel preparation procedure: One day prior to the procedure, the patient was placed on a liquid diet. A 10-hour preoperative fast from food and a 4-hour fast from water were implemented. Oral polyethylene glycol electrolyte powder was administered, with a total of three sachets, each dissolved in 1,000 mL of water. Two sachets were consumed between 19:00 and 21:00 on the evening before the procedure, and the remaining sachet was consumed between 4:00 and 5:00 on the morning of the procedure. Bowel preparation was considered adequate when clear, watery stools without fecal residue were achieved, ensuring complete bowel emptying.

FMT procedure: The bacterial suspension was rewarmed to 37 °C. A 50 mL aliquot of the suspension was administered via the nasojejunal tube using a syringe, followed by a flush with 10 mL of warm normal saline. After the infusion, the patient was positioned in the right lateral decubitus position for 30 min. The procedure was performed once every other day for a total of three transplantations.

### Patient 1

The patient was a male aged 4 years and 7 months. At the age of 3 years, the child exhibited developmental delays in language, social interaction, and cognitive abilities compared to peers. He was diagnosed with “childhood autism” at other hospital. In February 2025, he was admitted to the Department of Pediatrics, South China Hospital, Shenzhen University, to undergo FMT.

Past medical history: The patient had a history of food intolerance, characterized by recurrent gastrointestinal symptoms including abdominal pain, bloating, and diarrhea following the consumption of eggs, cow's milk, goat’s milk, and other foods, with a clear temporal association between symptom onset and food intake. Food allergen testing showed negative results for food-specific IgE. No organic gastrointestinal diseases were identified. There was no other significant medical history.

Birth and developmental history: The child was the first offspring of a full-term, uncomplicated vaginal delivery, with a birth weight of 2.9 kg. The mother's pregnancy was uneventful, and there was no history of perinatal hypoxia or asphyxia. The child achieved head control at 3 months, rolled over at 5 months, sat independently at 7 months, walked independently at 1.5 years, and began to say “mama” with intention at 1 year.

Physical examination: Height was 107 cm(P25-P50), weight was 16.9 kg(P25-P50), and the child was alert. Specialty examination: Motor function: Independent walking, running, hopping on one foot, and alternating feet while ascending/descending stairs were achieved. Speech and language: Spontaneous speech was limited; the child could produce short sentences of up to 10 words with unclear pronunciation. Social interaction and behavior: The child exhibited no eye contact or mutual gaze, showed no response when called by name, and was unable to establish peer relationships. Cognitive function: The child could identify facial features and common objects, and could understand and follow simple instructions. Stereotypic behaviors: No obvious stereotyped movements or restricted interests were observed.Activities of daily living: The child could drink from a cup and eat with his hands; he was non-compliant with dressing and unable to put on shoes independently; he could indicate the need to use the toilet but required assistance.Emotional presentation: The child was prone to crying and tantrums when demands were not met.

Family history: The parents were healthy and non-consanguineous, with no family history of psychiatric disorders or similar conditions. There was no family history of allergy-related diseases. No blood relatives within three generations had a history of delayed speech development.

Auxiliary examinations: The Childhood Autism Rating Scale (CARS) score was 35, and the Autism Behavior Checklist (ABC) score was 55. Food intolerance IgG testing [qualitative enzyme-linked immunosorbent assay (ELISA), Joinstar Biomedical Technology Co., Ltd.] revealed positive results for casein (+++), eggs (+++), milk (++), goat's milk (++), and sheep's milk (++). IgE testing was negative, and food-specific IgE antibodies were all negative.

Clinical management course: Prior to admission, the patient was advised to avoid foods associated with intolerance. Preoperative evaluations were completed to rule out contraindications for the procedure, and bowel preparation was performed. After bowel preparation, a nasojejunal tube was placed under colonoscopic guidance, and two hemostatic clips were used to anchor the disposable nasojejunal tube at the cecum, followed by FMT. Within three hours after FMT, the patient developed apathy, facial flushing, pruritus on both cheeks and ears, accompanied by abdominal bloating. Vital sign monitoring revealed increased respiratory and heart rates, while blood pressure remained within the normal range. Considering the overall clinical presentation, early-stage shock was suspected. Immediate fluid resuscitation with 10 mL/kg of normal saline administered intravenously over 15 min was initiated, along with oral cetirizine (2.5 mg) for anti-allergy treatment. Following prompt intervention, the patient's clinical symptoms completely resolved within 24 h. After symptom resolution, repeat laboratory tests showed normal complete blood count and C-reactive protein levels, with negative IgE results. Following resolution of the allergic reaction, FMT was temporarily discontinued, and the patient was observed for one day with stable condition. To reduce intestinal burden and enhance tolerance, the FMT bacterial solution dosage was adjusted to 25 mL per administration, administered once every 12 h for a total of four administrations, with no recurrence of adverse events.

At the one-month follow-up, the patient exhibited reduced abdominal pain, bloating, and diarrhea after consuming eggs and milk. In May 2025, the patient returned for a second course of FMT, which was completed without any adverse events. Repeat assessments showed a CARS score of 30 and an ABC score of 50. Improvements in core ASD symptoms included: Speech and language: The child demonstrated spontaneous verbal expression with his mother. Social interaction and behavior: The child exhibited active eye contact and mutual gaze with family members, responded when called by name, and was able to play with peers, although conflicts arose easily.

### Patient 2

The patient was a male aged 6 years. At the age of 3 years, the child exhibited significant developmental delays in language, social interaction, and cognitive abilities compared to peers, along with behavioral issues including destructiveness and impulsivity. He was diagnosed with “childhood autism” at other hospital. In August 2025, he was admitted to our hospital to undergo FMT.

Past medical history: The patient had a history of food intolerance, characterized by recurrent gastrointestinal symptoms including abdominal pain, bloating, and diarrhea following the consumption of eggs, milk, and other foods, with a clear temporal association between symptom onset and food intake. Food allergen testing showed negative results for food-specific IgE. No organic gastrointestinal diseases were identified. There was no other significant medical history.

Birth and developmental history: The child was the first offspring of a full-term, uncomplicated vaginal delivery, with a birth weight of 3.1 kg. The mother's pregnancy was uneventful, and there was no history of perinatal hypoxia or asphyxia. The child achieved head control at 3 months, rolled over at 5 months, sat independently at 7 months, walked independently at 11 months, and began to say “baba” and “mama” with intention at 1 year.

Physical examination: Height was 118.5 cm(P50-P75), weight was 17.9 kg(P3-P10), and the child was alert. Specialty examination: Motor function: Gross motor development was good, with the ability to perform coordinated actions such as running and jumping. Speech and language: The child had spontaneous speech and could produce long sentences of approximately 20 words, though pronunciation was unclear. Social interaction and behavior: Occasional eye contact and mutual gaze were observed; the child responded when called by name but had difficulty establishing effective peer relationships. Cognitive function: The child could understand and follow simple daily instructions and recognized common objects. Stereotypic behaviors: No obvious stereotyped movements or restricted interests were observed. Activities of daily living: The child was largely independent in daily living skills such as eating, drinking, and dressing. Emotional and attentional status: The child was generally emotionally stable but exhibited inattention.

Family history: The parents were healthy and non-consanguineous, with no family history of psychiatric disorders or similar conditions. There was no family history of allergy-related diseases. No blood relatives within three generations had a history of delayed speech development.

Auxiliary examinations: The CARS score was 32, and the ABC score was 54. Food intolerance IgG testing [qualitative enzyme-linked immunosorbent assay (ELISA), Joinstar Biomedical Technology Co., Ltd.] revealed positive results for eggs (++) and milk (+). IgE testing was negative, and food-specific IgE antibodies were all negative.

Clinical management course: Prior to admission, the patient was advised to avoid foods associated with intolerance. Preoperative evaluations were completed to rule out contraindications for the procedure, and bowel preparation was performed. After bowel preparation, a nasojejunal tube was placed under colonoscopic guidance, and two hemostatic clips were used to anchor the disposable nasojejunal tube at the cecum, followed by FMT. Ten hours after FMT, the patient developed generalized urticarial rash with pronounced pruritus and no other systemic symptoms. An acute allergic reaction was suspected, and oral cetirizine 10 mg was administered immediately for anti-allergy treatment, with rash resolution occurring one hour later. Twelve hours after FMT, the patient developed dizziness, severe abdominal pain, vomiting six times, and one episode of watery diarrhea. Physical examination revealed a respiratory rate of 40 breaths/min, pulse rate of 132 beats/min, blood pressure of 71/46 mmHg, lethargy, apathy, facial and lip pallor, tachypnea, muffled heart sounds, cool extremities, and a capillary refill time of 5 s. Anaphylactic shock was diagnosed. Immediate interventions included electrocardiographic monitoring, oxygen administration, fluid resuscitation with 20 mL/kg of normal saline administered intravenously over 10 min, and one intramuscular injection of 1:1,000 epinephrine (0.17 mg) into the lateral thigh. Ten minutes later, blood pressure was 70/41 mmHg, and a second dose of epinephrine (0.17 mg) was administered intramuscularly, followed by an additional fluid resuscitation with 20 mL/kg of normal saline administered intravenously over 10 min. Concurrently, methylprednisolone sodium succinate (2 mg/kg) was administered intravenously via dual access for anti-inflammatory therapy. Ten minutes later, repeat blood pressure was 92/53 mmHg, heart rate was 92 beats/min, and cardiac auscultation revealed strong, regular heart sounds.Following the comprehensive resuscitation efforts, the patient's dizziness, abdominal pain, and vomiting resolved within 50 min, heart rate and blood pressure normalized, and peripheral circulation improved. All symptoms completely resolved within 24 h. After symptom resolution, follow-up laboratory tests revealed no significant abnormalities on complete blood count, normal C-reactive protein levels, and positive total IgE. Respiratory pathogen testing and stool pathogen testing showed no abnormalities. Following resolution of the allergic reaction, the patient was hospitalized for an additional three days of observation and was discharged after achieving clinical stability. Due to the severe allergic reaction, the FMT course was not completed.

At the one-month follow-up, the patient had no recurrence of allergic symptoms.

The chronological course of pretreatment, FMT procedures, adverse reactions, emergency management, follow-up outcomes and subsequent treatment adjustments for these two ASD children with multiple food intolerances are summarized in Table 1.

Donor information: The fecal microbiota suspension was provided by Shenzhen Qiyuan Future Biotechnology Co., Ltd., sourced from a 10-year-old healthy male (batch number GZ001). Five days prior to stool donation, the donor underwent dietary restrictions to avoid foods to which the recipient was allergic or intolerant. The donor tested negative for common allergens. The donor's gut microbiota composition was assessed using 16S rRNA high-throughput sequencing. Microbiota diversity was similar to that of healthy children of the same age, with no significant dysbiosis and a relatively stable community structure. At the phylum level, Bacteroidetes was the dominant phylum, and no pathogenic bacteria were detected. The donor met the general screening safety standards for pediatric fecal microbiota transplantation.

## Discussion

The therapeutic value of FMT in the management of pediatric ASD has received increasing attention. It is currently believed that FMT alleviate neuroinflammation and promote behavioral improvements by modulating the gut–brain axis, reducing pro-inflammatory cytokine levels, and increasing the production of beneficial metabolites such as short-chain fatty acids (9).However, larger, rigorously designed clinical and basic studies are still needed to confirm the therapeutic efficacy. A review of adverse events associated with FMT over the past two decades reported that common adverse reactions included transient diarrhea, abdominal pain, and bloating, with a serious adverse event rate of 0.9%, suggesting an overall favorable safety profile (3). Meta-analysis data have shown that the adverse event rate of FMT is 9.3%, with the majority being mild and self-limiting (10). A single-center retrospective study analyzing adverse events in 114 patients who underwent FMT concluded that most post-FMT adverse events were transient, self-limiting, and manageable, with only one case of a patient with primary immunodeficiency who received FMT for chronic refractory diarrhea and died of sepsis and liver failure four weeks later (11). However, among 84 children with ASD who received FMT at our center during the same period, only two cases with comorbid multiple FIs developed anaphylactic shock. To the best of our knowledge, this is the first report in the pediatric field of FMT-related severe anaphylactic shock in children with ASD and comorbid FI. This finding directly challenges existing perceptions and provides the first clinical evidence for safety assessment of FMT in this high-risk subgroup, filling a gap in current diagnostic and treatment guidelines regarding risk stratification for this specific population.

Case 1 presented with early-stage shock. Considering the patient's multiple positive food-specific IgG antibodies and the clinical basis of impaired intestinal barrier function, we hypothesize that the shock may have been associated with IgG-mediated type III hypersensitivity (12). The proposed mechanism is as follows: components of the donor microbiota or the transplant suspension may have entered the circulation through the compromised intestinal barrier and formed IgG–antigen complexes. These immune complexes can activate the complement system (C3a/C5a) and Fc*γ* receptors, recruiting neutrophils to release proteases, thereby inducing cutaneous vascular endothelial inflammation and tissue injury (13). When the complement system is overactivated, excessive production of C3a and C5a can persistently stimulate mast cells to release inflammatory mediators, leading to systemic vasodilation and increased vascular permeability, resulting in reduced effective circulating blood volume, hypotension, and ultimately shock (14). However, complement components such as C3a and C5a were not measured in this study, and direct laboratory evidence supporting the above mechanism is lacking. Therefore, this mechanistic hypothesis remains a theoretical inference based on clinical manifestations and has not been directly validated, which constitutes one of the limitations of this study.

In Case 2, urticaria developed several hours after FMT and resolved following antihistamine treatment. Approximately two hours later, systemic symptoms appeared and progressed to shock. This clinical course was characterized by relapse after initial resolution without re-exposure to the trigger, raising the possibility of biphasic anaphylaxis (15). According to the 2020 World Allergy Organization diagnostic criteria for anaphylactic shock (16), this patient had acute onset with involvement of both the skin/mucosal system and the circulatory system (hypotension), meeting the diagnostic criteria for anaphylactic shock. Further analysis suggests that the donor suspension may have contained histamine-producing bacteria (e.g., Escherichia coli, Proteus species), leading to increased histamine production. Histamine is rapidly absorbed from the gut into the circulation, activating mast cells to release inflammatory mediators, thereby triggering cutaneous allergic reactions and vasodilation (17). Histamine also acts on vascular smooth muscle, resulting in fluid extravasation and hypotension, characteristic manifestations of shock. On the other hand, the possibility that post-FMT gut microbiota remodeling triggers an abnormal immune response cannot be excluded and requires further investigation.

Integrating the pathogenesis of the two cases, the shared pathological basis of “gut microbiota dysbiosis – impaired intestinal barrier function – immune dysregulation” in children with ASD and FI may represent the intrinsic predisposition for a robust immune response to FMT-related stimuli. It may also explains why severe anaphylactic shock occurred only in the ASD with FI subgroup rather than in children with ASD alone.

Furthermore, in this study, both adverse events occurred within a short period after completion of the FMT procedure, rather than after food intake, after endoscopic manipulation, or after administration of anesthetic agents, and no procedure-related complications such as bleeding, perforation, or infection were observed. The temporal relationship and clinical presentation suggest that the adverse events were related to FMT, potentially associated with the transplanted donor microbiota, components of the transplant suspension, microbial metabolites, or bacterial proteins. In Case 1, no similar severe reaction occurred during subsequent FMT procedures; the specific reasons for this remain unclear and may be related to multiple factors, including gradual adaptation of the intestinal immune status and restoration of the intestinal barrier. Due to the lack of relevant laboratory testing, these issues could not be further explored, which also highlights the need for more systematic assessment and long-term observation of allergic risks associated with FMT infusion.

Based on the experience of resuscitation and subsequent management of these two cases, we found that post-FMT anaphylactic shock has a distinct progressive pattern. In Case 1, pruritus and facial flushing appeared first, while in Case 2, generalized urticaria was the initial manifestation. These cutaneous manifestations were all prodromal symptoms of shock, indicating that skin-related symptoms should be regarded as high-risk warning signs after FMT rather than mild adverse reactions, and immediate evaluation should be initiated. In the resuscitation phase, both children achieved stabilization through standardized management: Case 1 received early fluid resuscitation combined with antihistamine therapy, and Case 2 received intramuscular epinephrine, dual-access fluid resuscitation, and corticosteroid therapy. These experiences confirm that standardized pediatric anaphylactic shock emergency protocols are applicable to FMT-related adverse events. For subsequent treatment adjustments, Case 1 received a low-dose regimen (25 mL per administration) with shortened intervals, which effectively improved tolerance and provides a specific dosing reference for optimizing FMT protocols in high-risk children.

Important implications from this study include the following. For the high-risk population of children with ASD and comorbid FI, refined preoperative screening and risk stratification are essential: complete serum food-specific IgE testing, obtain a detailed allergy history, and clarify whether there is a history of recurrent gastrointestinal symptoms associated with the ingestion of specific foods. In donor selection, priority should be given to donors with no family history of food intolerance, no histamine-producing bacteria in microbiota testing, and negative allergen screening. Parents must be fully informed of the potential risk of FMT-related anaphylactic shock when FI is present, and specific informed consent should be obtained. During the procedure, standardized operations and emergency preparedness should be implemented: resuscitation equipment including epinephrine, corticosteroids, and oxygen should be readily available at the bedside, and the medical team should be familiar with the emergency protocol for FMT-related anaphylactic shock to ensure rapid response. Post-procedure monitoring should be targeted: in addition to routine vital signs (blood pressure, heart rate, respiration), cutaneous manifestations (pruritus, rash, flushing) should be considered core monitoring indicators and assessed every 30 min. Regarding observation duration, such children should be monitored for at least 24 h after FMT to ensure post-procedural safety.

This study has several limitations. First, this study is a single-center, small-sample case report without quantitative statistics or control analysis, and all conclusions are observational in nature, so the generalizability of the findings is limited. Second, key indicators such as complement components C3a/C5a, histamine, and microbial metabolites were not measured, precluding direct validation of the hypersensitivity mechanisms. Third, the follow-up duration was short, and long-term safety and efficacy data are lacking. Future large-sample or prospective studies are needed to further clarify the relevant mechanisms. Fourth, the diagnosis of food intolerance mentioned in this article is only a presumptive diagnosis based on clinical history and symptoms, rather than a confirmed diagnosis verified by the double-blind placebo-controlled food challenge.

In conclusion, while FMT has potential therapeutic value for children with ASD, clinicians should be highly vigilant about the risk of severe anaphylactic shock when applying FMT to the specific population of children with ASD and comorbid FI. Based on the refined risk assessment, close monitoring throughout the procedure, and standardized emergency management strategies proposed in this study, clinicians can maximize patient safety while exploring the therapeutic potential of FMT, thereby providing a better basis for the clinical management of this special population.

## Data Availability

The datasets presented in this article are not readily available because the original detailed medical records involve minors' private health information and are prohibited from public deposition in any database per hospital ethics rules. Requests to access the datasets should be directed to the corresponding author.
